# Alcohol’s Harmful Effects on Bone

**Published:** 1998

**Authors:** H. Wayne Sampson

**Affiliations:** H. Wayne Sampson, Ph.D., is a professor in the Department of Human Anatomy & Medical Neurobiology, Texas A&M Health Science Center, College of Medicine, College Station, Texas

**Keywords:** chronic AODE (alcohol and other drug effects), bone, bone fracture, adolescent, adult, metabolism, biochemical mechanism, hormones, parathyroid, vitamin D, calcitonin, growth promoting factors, cell type, moderate AOD use, female, literature review

## Abstract

Long-term alcohol consumption can interfere with bone growth and replacement of bone tissue (i.e., remodeling), resulting in decreased bone density and increased risk of fracture. These effects may be exerted directly or indirectly through the many cell types, hormones, and growth factors that regulate bone metabolism. Alcohol consumption during adolescence reduces peak bone mass and can result in relatively weak adult bones that are more susceptible to fracture. In adults, alcohol consumption can disrupt the ongoing balance between the erosion and the remodeling of bone tissue, contributing to alcoholic bone disease. This imbalance results in part from alcohol-induced inhibition of osteoblasts, specialized cells that deposit new bone. Some evidence suggests that moderate drinking may decrease the risk of fracture in postmenopausal women.

Bone is a living tissue that continues to undergo change and replacement (i.e., remodeling) even after a person has attained full stature. Long-term alcohol consumption can interfere with bone growth and remodeling, resulting in decreased bone density and increased risk of fracture. Those effects may be exerted directly or indirectly through the many cell types, hormones, and growth factors that regulate bone metabolism. This article summarizes alcohol’s harmful effects on bone in both adults and adolescents. In addition, the article suggests possible physiological mechanisms that account for the development of those harmful effects.

## Bone Structure and Growth

**Figure 1:**
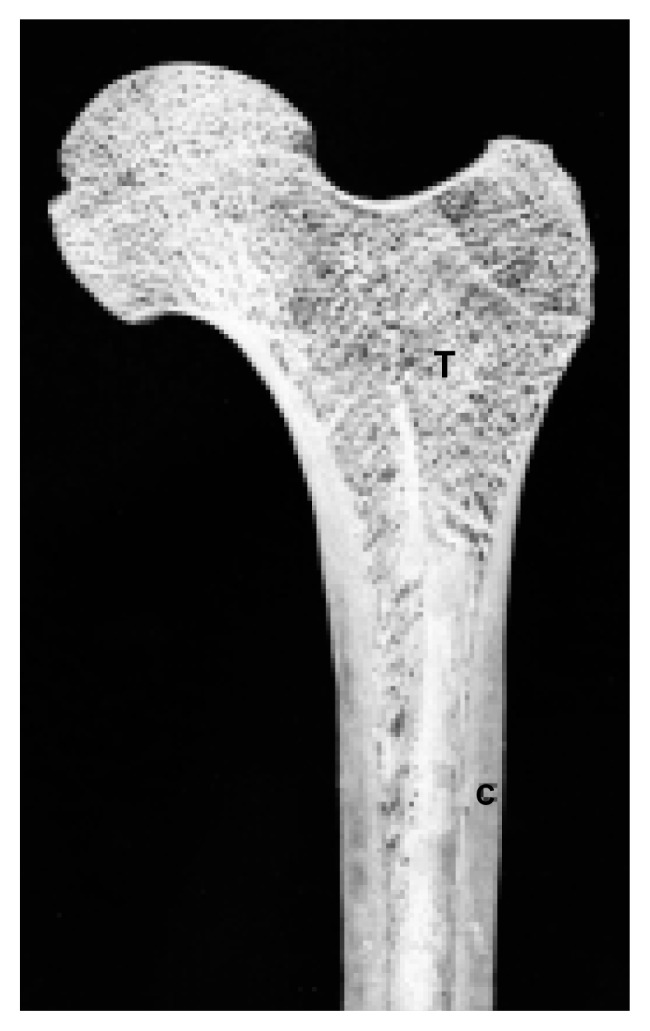
A section of the upper end of the long bone of the thigh (i.e., the femur). The ball at the upper left forms part of the hip joint. The walls of the shaft (C) are mainly cortical bone; the bone nearer the ends (T) is cancellous bone.

**Figure 2:**
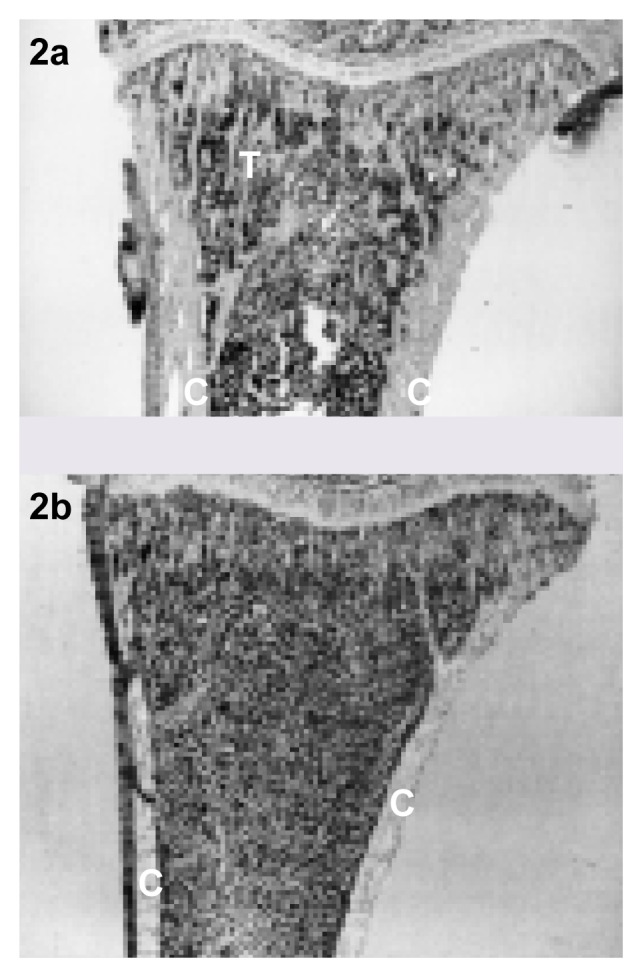
The effect of alcohol on the microscopic structure of long bones in the rat. The sections show the upper end of the tibia, the lower leg bone that forms part of the knee joint. Compared with normal bone (2a), tibiae from rats administered alcohol (2b) exhibit more compact cortical bone (C) and significantly less cancellous bone. T indicates a single trabeculum, one of the structural units of cancellous bone.

Bone consists of living cells encased in a hard matrix of protein fibers and calcium crystals. There are two types of bone. Cortical bone, which is dense and thick, forms the outer layer of bones and the shafts of the long bones of the arms and legs (see figure 1). Cancellous bone, which is a porous meshwork of thin plates (i.e., trabeculae), occurs mostly within the ends of long bones and in the vertebrae. Alcohol affects both types of bone, although the most dramatic changes occur in cancellous bone (see figure 2, p. 192).

The process of skeletal growth and maturation involves three general phases: (1) growth and modeling, (2) consolidation, and (3) remodeling. Bones grow rapidly from birth until the end of puberty. After bones stop growing lengthwise, they continue to increase in mass (referred to as consolidation) (Matkovic 1991), probably reaching peak bone mass at some point between ages 9 and 18 (Matkovic 1991). From age 16 throughout adulthood, bone undergoes constant remodeling and replacement in order to maintain resilience and adapt to shifting stress patterns (Kanis 1994).

Between ages 20 and 40, bone density begins to decline, resulting in a cumulative decrease in skeletal mass of 30 to 40 percent by age 70 (Moran et al. 1995). Women experience an accelerated reduction in bone mineral density following menopause (as discussed in the section “Reproductive Hormones,” p. 192) (Kanis 1994). As aging bones weaken, they reach a point (i.e., fracture threshold) at which even minor stress can cause fractures.

## Adolescent Bone Development and Alcohol

Achieving an optimal peak bone mass during adolescence may reduce a person’s risk for developing osteoporosis (i.e., bone loss with fracture) later in life. A high peak bone mass should withstand a longer duration and greater level of bone loss before reaching the fracture threshold. Although peak bone mass appears to be largely under genetic control (Pocock et al. 1987), it can be influenced by hormonal, nutritional, environmental, and lifestyle factors, including tobacco and alcohol consumption.

A significant proportion of the adolescent population may be at risk for alcohol’s harmful effects on bone. A nationwide survey of more than 50,000 high school students found that 63 percent of seniors had been drunk at least once and 51 percent had consumed alcohol in the month before the survey. Most respondents had consumed alcohol for the first time before age 13 (Arria et al. 1991).

Results of experiments using laboratory animals suggest potential consequences of alcohol consumption during adolescent bone growth. Long-term alcohol administration to young, rapidly growing rats significantly reduced bone growth, volume, density, and strength (Hogan et al. 1997; Sampson et al. 1996, 1997). The longitudinal growth rate and the rate of proliferation of cells in the growing region near the ends of long bones (i.e., growth plates) stop during long-term alcohol administration. If those effects occur in humans, they could significantly decrease bone mass. The decreased bone mass that occurs from early, long-term alcohol consumption could result in increased fracture and early onset of osteoporosis.

## Alcohol’s Effects on Hormones that Regulate Bone

In addition to providing structural support, bone is a major storage depot for calcium and other minerals. The small intestine absorbs calcium from ingested food, and the kidneys excrete excess calcium. An adequate concentration of calcium in the bloodstream is required for the proper functioning of nerves and muscle. The body monitors calcium concentration and responds through the action of hormones, vitamins, and local growth factors to regulate the distribution of calcium between blood and bone. Alcohol may disrupt this balance by affecting the hormones that regulate calcium metabolism as well as the hormones that influence calcium metabolism indirectly (e.g., steroid reproductive hormones and growth hormone [GH]) (Sampson 1997).

### Calcium-Regulating Hormones

#### Parathyroid Hormone

**Figure 3:**
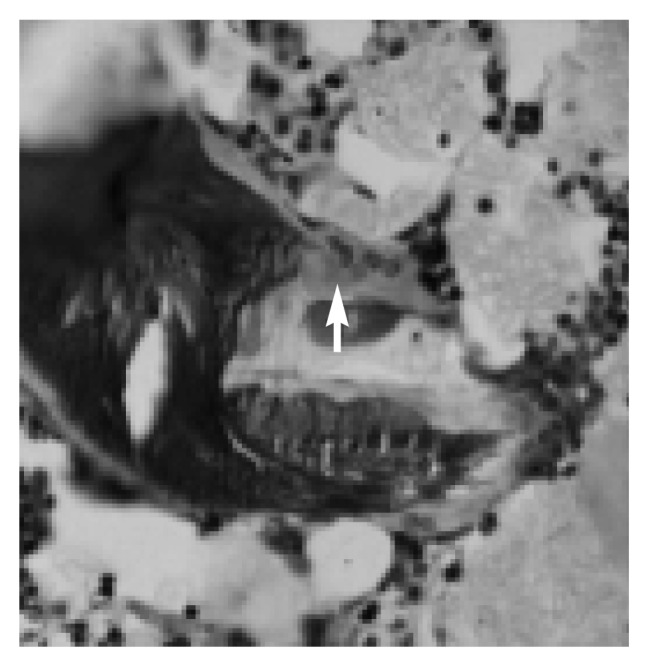
Microscopic view of a bone-resorbing cell, or osteoclast (see arrow).

Parathyroid hormone (PTH) is secreted into the bloodstream by four small glands located behind the thyroid gland in the neck. The hormone, which is produced in response to decreasing levels of calcium in the blood, stimulates the activity of specialized bone cells called osteoclasts (see figure 3, p. 193). Osteoclasts dissolve small areas of bone, releasing calcium into the blood. (The role of osteoclasts in bone remodeling is discussed in the section “Alcohol’s Effects on Bone Remodeling,” p. 193) In addition, PTH inhibits the excretion of calcium by the kidney and activates vitamin D, which promotes the absorption of calcium from the intestine. The resulting increase in calcium levels eventually inhibits further PTH production.

Short-term alcohol consumption increases PTH secretion, possibly by causing calcium to leave body fluids (e.g., blood) and flow into cells (Williams et al. 1978). Laitinen and colleagues (1991) administered intoxicating doses of alcohol over a 3-hour period to men and women who were moderate drinkers, with each person receiving approximately 5 to 11 standard drinks.^1^ Levels of PTH declined sharply until the end of the drinking period and rose over the next 9 hours, eventually exceeding levels measured before alcohol consumption. Urinary calcium excretion increased during the first 3 hours and subsequently decreased. Long-term heavy drinking was associated with low blood calcium (i.e., hypocalcemia) but normal PTH levels (Laitinen et al. 1991).^2^ Those findings indicate that the hypocalcemia did not result from reduced PTH secretion and also suggest that alcohol administration impaired the ability of the parathyroid glands to increase PTH production in response to the presence of hypocalcemia (Laitinen et al. 1994).

#### Calcitonin

Specialized cells in the thyroid gland produce calcitonin, a hormone that protects the skeleton from calcium loss by inhibiting osteoclast activity. In contrast to the action of PTH, calcitonin increases the deposition of calcium in bone and lowers the level of calcium in the blood. Calcitonin levels increase only briefly during acute and short-term alcohol consumption (Balabanova et al. 1989; Williams et al. 1978). The significance of this effect is uncertain.

#### Vitamin D

Vitamin D increases intestinal absorption of dietary calcium and has a function in normal bone metabolism. Vitamin D is formed in the skin through the action of sunlight and occurs in foods such as liver, eggs, and milk. The vitamin becomes physiologically active only after chemical modification in the liver and kidneys. Alcoholics normally have low levels of activated vitamin D, along with low levels of the proteins that bind with vitamin D to protect it during transport within the blood (Bikle et al. 1993). Vitamin D levels are especially low in the presence of alcoholic liver disease (e.g., alcoholic cirrhosis). The alcohol-induced decrease in activated vitamin D results in decreased absorption of calcium, although calcium levels quickly return to normal following abstinence (Krawitt 1975).

### Reproductive Hormones

Osteoporosis can develop in post-menopausal women (Kanis 1994) as well as in men with inadequate gonadal function (Jackson et al. 1987). Alcoholic men frequently have decreased levels of the male steroid hormone testosterone (produced mainly in the testes), and female alcoholics experience increased metabolic conversion of testosterone (produced in the ovaries and adrenal glands) to the female steroid hormone estradiol (Diamond et al. 1989; Gavaler and Van Thiel 1992). Because estrogen deficiency is a major contributing factor for the development of osteoporosis, alcohol might indirectly affect bone through estrogen. Estrogen replacement reduces a woman’s risk of developing postmenopausal osteoporosis. In addition, moderate alcohol consumption has been reported to increase estrogen levels in the blood. A review of published research on alcohol and estrogen (Purohit 1998), however, concluded that moderate alcohol consumption (i.e., no more than one drink per day for women) does not appear to have a significant effect on levels of estradiol, the most potent of the estrogens.

### Effects of Moderate Alcohol Consumption

Studies show a relationship between the consumption of large quantities of alcohol and bone loss. However, a few studies indicate that moderate alcohol consumption may help reduce osteoporosis and decrease fracture risk in postmenopausal women (Holbrook and Barrett-Connor 1993; Laitinen et al. 1991). For example, in a study of more than 14,000 subjects, Naves Diaz and colleagues (1997) reported that women age 65 and older who consume alcohol on more than 5 days per week had a reduced risk of vertebral deformity compared with those who consumed alcohol less than once per week.

Two recent studies investigated the influence of moderate alcohol consumption on rats following surgical removal of their ovaries (i.e., ovariectomy) to mimic menopause. In one study (Fanti et al. 1997), rats were administered doses of alcohol equivalent to 18 percent of their total dietary caloric intake for 3 weeks. Sampson and Shipley (1997) administered the equivalent of two standard drinks, as previously defined, per day for 6 weeks. In both studies, ovariectomized rats exhibited decreased bone density and bone volume compared with nonovariectomized rats. However, these changes were not significantly affected by alcohol administration. Although Fanti and colleagues (1997) found fewer osteoclasts in ovariectomized alcohol-fed animals, this finding was not reflected in decreased bone volume. Therefore, neither study identified a beneficial effect of moderate alcohol consumption on bone quality.

### Growth Hormone

Growth hormone, secreted by the pituitary gland, is important in bone growth and remodeling. Growth hormone exerts its effects largely through a hormone called insulin-like growth factor 1 (IGF-1), which is produced in the liver and other organs (see article by Dees and colleagues, pp. 165–169). Levels of IGF-1 are significantly reduced in alcohol-fed animals until 7 months of age (Sampson et al. 1998).^3^ The aforementioned findings might explain the greatly reduced rates of longitudinal growth and the proliferation of certain cell types in the growth plates of young, rapidly growing animals (Sampson et al. 1997). The levels of IGF-1 in alcohol-fed animals become normal after 6 months of alcohol feeding, but bone deficiencies resulting from alcohol consumption continue, possibly through a mechanism independent of growth factors.

## Alcohol’s Effects on Bone Remodeling

**Figure 4:**
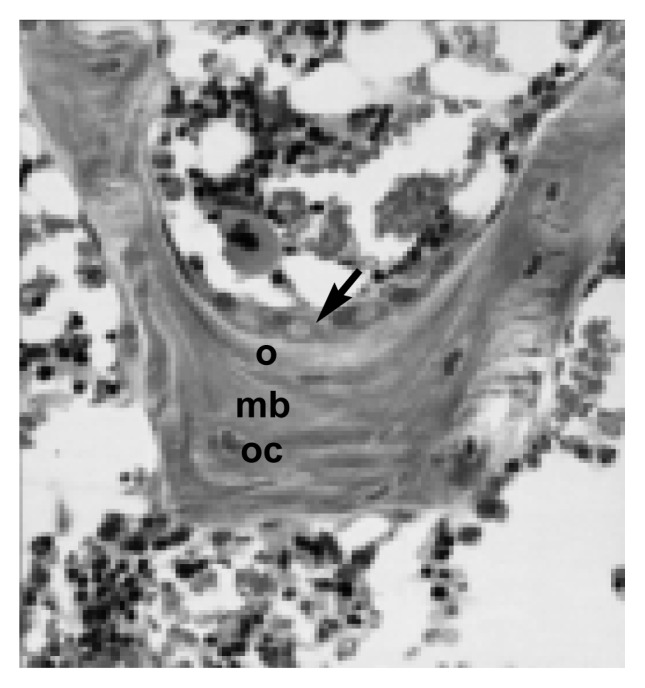
Microscopic view of bone-forming cells, or osteoblasts (arrow), showing a newly formed layer of unmineralized bone matrix (o) and an older layer of mineralized bone (mb). Occasionally, osteoblasts become embedded within the mineralized matrix, losing their bone-forming ability (oc).

Remodeling occurs in small, circumscribed areas scattered on the surface of the bone. Osteoclasts (see figure 3) erode a cavity on the bone surface in a process known as resorption. When the resorption cavity is complete, the osteoclasts disappear, the floor of the cavity is smoothed off, and a thin layer of matrix or cement is deposited. Bone-forming cells (i.e., osteoblasts) (see figure 4) fill the newly formed cavity with new bone. Local imbalance of bone remodeling can occur when osteoclasts erode cavities that are too deep or when osteoblasts lay down layers of new bone which are too shallow. It is unclear whether alcohol’s effects on bone remodeling result from improper bone formation or overactive osteoclast resorption.

Schnitzler and Solomon (1984) found that alcohol administration reduced bone formation and increased bone resorption. In a study of men who were daily drinkers and who had osteoporosis of unknown origin, De Vernejoul and colleagues (1983) found a markedly reduced mean wall thickness (i.e., the thickness of the newly formed structural unit formed during remodeling), which they took into account when measuring the quantity of resorbed bone. Based on their calculations, De Vernejoul and colleagues theorized that alcoholic osteoporosis is characterized by decreased bone formation but normal levels of resorption. A microscopic analysis of bone tissue from men with osteoporosis (Chappard et al. 1991) confirmed that alcohol consumption leads to delayed and impaired osteoblast activity associated with normal osteoclast function.

To determine whether alcohol has a direct effect on osteoblasts, researchers measured levels of osteocalcin, a protein secreted by osteoblasts and thought to be a measure of osteoblast function. Using in vitro preparations of osteoblasts from rats, most investigators reported a decrease in osteocalcin levels in response to alcohol administration, suggesting that alcohol decreases osteoblastic activity (Peng et al. 1991). Microscopic studies of bone tissue from rats demonstrated decreased trabecular bone volume, decreased numbers of osteoblasts, and decreased rates of bone formation, indicating impaired bone formation and mineralization, along with other characteristics indicative of osteoporosis (Sampson 1998; Sampson et al. 1997). In addition, the amount of trabecular surface covered by active osteoblasts was significantly reduced in alcohol-fed rats, suggesting an inhibition of osteoblast proliferation. Wall thickness, another measure of osteoblast activity, was reduced by 52 percent in alcohol-fed animals compared with animals not administered alcohol (Dyer et al. 1998). These findings agree with in vitro studies that demonstrate diminished osteoblast numbers and osteoblast function in humans (Klein et al. 1996; Chavassieux et al. 1993; Friday and Howard 1991). Overall, alcohol appears to suppress osteoblast function in adults, resulting in decreased bone formation.

## Summary

Chronic alcohol consumption has major harmful effects on bone development and maintenance at all ages. Alcohol’s action on young, growing bone is especially deleterious, because alcohol reduces peak bone mass and can result in relatively weak adult bones that are more susceptible to fracture. Alcohol and bone effects modulate and are modulated by hormones, including PTH, calcitonin, and growth hormone, as well as by other substances, such as vitamin D. However, these interactions do not appear to be the major mechanism of alcohol’s effects on adult bone. Results of human and animal experiments indicate that alcohol directly inhibits the action of bone-forming cells. Alcohol’s effect on bone-resorbing cells, however, is uncertain. Additional research is needed to explain all of alcohol’s effects on bone.

## Footnotes

1 Moderate drinking is generally defined as no more than two standard drinks per day for men and one per day for women. A standard drink is generally considered to be 12 ounces of beer, 5 ounces of wine, or 1.5 ounces of 80-proof distilled spirits (National Institute on Alcohol Abuse and Alcoholism 1992).

2 One-half of the subjects in this study consumed more than 10 drinks per day, more than one-half had been drinking heavily for at least 5 years, and the median duration of the most recent drinking bout was 9 days (maximum 180 days). The average blood alcohol concentration was 0.3 percent, *at least* three times the legal limit for driving in any State.

3 This age is approximately equivalent to the mid-thirties in a human.
